# Short-term ingestion of sublethal microcystin levels disrupts stress response in male mice

**DOI:** 10.3389/fendo.2025.1568923

**Published:** 2025-05-26

**Authors:** Salan Ghaju, Evance Pakuwal, Rafaela S. C. Takeshita, Xiaozhen Mou, Wilson C. J. Chung

**Affiliations:** ^1^ School of Biomedical Science, Kent State University, Kent, OH, United States; ^2^ Department of Medical Sciences, University of Adelaide, Adelaide, SA, Australia; ^3^ Department of Anthropology, Kent State University, Kent, OH, United States; ^4^ Department of Biological Sciences, Kent State University, Kent, OH, United States

**Keywords:** cyanobacterial harmful algal bloom, microcystin, brain, stress, hypothalamus, paraventricular nucleus, hippocampus, inflammation

## Abstract

Cyanobacterial harmful algal blooms (cHABs) produce various cyanotoxins, primarily microcystin leucine arginine (MC-LR). Studies demonstrated that intraperitoneal delivery of high-dose MC-LR affects the rat stress response, which is regulated by the hypothalamus-pituitary-adrenal (HPA) axis. In general, during stress parvocellular neurons in the hypothalamic paraventricular nucleus (PVN) secrete corticotropin-releasing hormone (CRH), triggering adrenocorticotropic hormone (ACTH) release from the anterior pituitary, which leads to increased cortisol in humans and corticosterone in rats and mice. Here, we tested the hypothesis that short-term sublethal ingestion of MC-LR activates the peripheral and central components of the HPA axis. First, we found that young adult male mice gavaged with MC-LR (50 μg/kg bw, p.o.) every 2 days for 21 days had elevated plasma corticosterone levels when compared to H_2_O (vehicle) mice. Ingestion of contaminated fresh water is a likely natural route of MC-LR exposure for animals, including humans. Second, *Crh* and arginine vasopressin (*Avp*) mRNA expressions were elevated in the hypothalamus of MC-LR-dosed mice. Third, ΔFOSB (i.e., long-term cell activity marker) immunofluorescence in the PVN and hippocampal dentate gyrus (DG) of MC-LR mice was significantly elevated compared to vehicle mice, but not in cornu ammonis (CA) 1, 2 and 3. In contrast, MC-LR mice had reduced hypothalamic glucocorticoid receptor (*Gr*) mRNA expression. Fourth, no significant changes were found in the mRNA expression of the inflammatory markers: tumor necrosis factor α (*Tnf-α*) and interleukin-1β (*IL-1β*) in the hypothalamus, liver, and spleen and C-reactive protein (*Crp*) in the liver and spleen. These data indicate that short-term ingestion of sublethal levels of MC-LR resulted in increased peripheral and central HPA axis activity.

## 1 Introduction

Anthropogenic activities induced water eutrophication (i.e., increased levels of nitrogen and phosphorus), and higher water temperatures have significantly increased the frequency of cyanobacterial harmful algal blooms (cHABs) in freshwater (1). This has dramatically elevated the production and release of hazardous cyanotoxins, mainly microcystins (MCs) (2–4). Current climate models predict that cHABs will persist and expand in freshwater (5). Moreover, while water treatment plants can remove MCs, effective removal is fiscally not feasible (6). Therefore, accidental and short-term exposure to environmental levels of MCs is almost inevitable through ingestion, inhalation, and dermal contact with MC-contaminated drinking water. For example, in the summer of 2014, MC levels exceeded 1 µg/L in Toledo, OH drinking water due to cHABs in Western Lake Erie, OH (6). This single event led to an immediate two-day ban on local drinking water usage, which left half a million residents without a source of clean public drinking water at a substantial economic cost. More importantly, from a public health point of view, exposure to MC-contaminated water in high concentrations has been shown to cause acute liver toxicity (7–10) resulting in apoptotic or necrotic cell death (10, 11) or even mortality in humans (12).

There are more than 200 MC congeners, of which MC leucine arginine (MC-LR) is the most prevalent, best-studied, and one of the most toxic variants (13–16). Overall, environmental MC-LR levels in freshwater fluctuate highly between locations (from undetectable to over 1000 µg/L) (2, 3, 17–19). Ingested MC-LR is primarily passively absorbed by the small intestines into the portal vein and transported through the blood from the gut to other organs. Absorbed MCs are quickly taken up by mammalian cells due to their water-solubility and the presence of transmembrane organic anion transport polypeptides (OATPs), which have been widely detected in various cell types, including the liver, kidney, intestines, and brain (8, 20–22). Following absorption through gut, the MC-LR is passed into the liver which then goes into the blood stream and then to other organs (23, 24). Also, exposure to MC-contaminated water in high concentrations has been shown to cause acute liver and splenic toxicity (7–10, 25, 26). Microcystin-LR is a well-known covalent inhibitor of protein phosphatase 1 (PP1) and protein phosphatase 2A (PP2A). Inhibition of the major cellular dephosphorylating enzymes can lead to cellular dysfunction and apoptosis (27–30).

There is evidence indicating that exposure to intraperitoneal MC-LR induces toxicity that impacts neuroendocrine functions that control the mammalian stress response (31–34). In general, the mammalian stress response is regulated by the hypothalamus-pituitary-adrenal (HPA) axis. Stressors activate hypothalamic PVN parvocellular neurons to release CRH, which stimulates ACTH release from the pituitary, and subsequent glucocorticoids release from the adrenals: cortisol in humans and corticosterone in rodents. Upon cessation of the stressor, glucocorticoids provide negative feedback to the pituitary, hypothalamus, and hippocampus to inhibit HPA activation and return to homeostatic physiological conditions (35–42). In addition, in rodents, repeated stress was shown to also induce AVP expression in CRH PVN neurons to potentiate CRH-dependent HPA activation (37, 38, 43, 44). We and others have shown that disrupted HPA activity is linked to promoting anxiety and depression (39, 45–49).

Previous studies showed that concentration, exposure route, and exposure length dictate MC-LR toxicity. For example, a single intraperitoneal injection of MC-LR in male and female rats reduced HPA activity 24 hours later (33, 34). In contrast, HPA activity was elevated in rats that received daily intraperitoneal MC-LR dosing for 42 days (50). Similarly, zebrafish immersed in MC-LR contaminated water (as low as 1 µg/L) for 30 days caused an activated stress axis (51). Moreover, the LD50 of intraperitoneal MC-LR exposure is around 50 µg/kg (16, 52), which contrasts oral MC-LR toxicity of 30–100 times less (16). Therefore, the route and length of MC-LR exposure are critical for determining its toxicity to mammalian HPA function.

In this study, we investigated the impact of short-term (< 1 month) oral MC-LR ingestion rather than intraperitoneal injections at sublethal levels on the stress axis, which has not been studied in depth. Young adult male C57BL6 mice (2 months of age) were gavaged every two days with MC-LR for 21 days. Specifically, we investigated MC-LR effects on the hypothalamic cellular activation in the context of the stress response and the peripheral inflammatory response. We found that short-term oral ingestion of sublethal MC-LR levels elevated central and peripheral HPA activity without significant activation of the central and peripheral inflammatory response markers. Furthermore, there was evidence that the HPA negative feedback was disrupted in animals treated with MC-LR. These results indicated that MC-LR caused a maladaptive HPA response that may contribute to stress-related mental health conditions, such as anxiety and depression (39, 45, 46).

## 2 Materials and methods

### 2.1 Animals

Male C57BL/6 mice (6 weeks of age) were obtained from the Jackson Laboratories (Bar Harbor, ME). Mice were kept in an animal room and held at 22-25°C and 50-65% relative humidity with a 12-hour light/dark cycle. The mice were given tap water, standard pellet food (Prolab RHM 3000, Lab Diet, St. Louis, MO), *ad libitum*. The Institutional Animal Care and Use Committee (IACUC # 525 WC 22-03) approved all procedures.

### 2.2 MC-LR ingestion

C57BL/6 mice were habituated for 2 weeks and were randomly divided into 2 groups: vehicle (n = 9) and MC-LR (n = 6) treatment groups were determined by power analysis (i.e., significance level of 0.05, power of 0.8 and enrollment of 1.5 favoring vehicle was used to obtain additional vehicle gavaging data). The body weight of the animals was measured every day. Microcystin-LR (101043-37-2, Cayman Inc., Ann Arbor, MI) stock solution of 10 mg/ml was diluted into a working stock of 12.5 μg/ml with sterile water and stored at -20°C, which was used to gavage mice using a 30 mm flexible oral polypropylene feeding tube (FTP-20-30, Instech Labs Inc, Plymouth Meeting, PA) with MC-LR at 50 μg/kg body weight. Based on average daily water consumption of C57BL6 mice (53), 50 µg/kg is equivalent to approximately 187 µg/L, a value this is not only readily found in MC-LR contaminated freshwater sources, but also far lower than the documented ingested MC-LR lethal dose 50 (LD50) (16) or lowest observed adverse effect levels (LOAELs) of 5 mg/kg (15). The vehicle mice were gavaged with equivalent sterile water volume based on 50 µg/kg MC-LR. All mice were treated every 48 hours for 21 days. On the 22^nd^ day, mice were deeply anesthetized with isoflurane and rapidly decapitated.

### 2.3 Blood collection and brain tissue collection

Trunk blood was collected in tubes coated with 0.5 M EDTA and kept on ice before centrifugation at 1000 x *g* at 4°C for 10 minutes. The plasma was stored at -20°C. Following, the brain was retrieved from the skull and divided sagitally into two hemispheres. One half was placed in 4% paraformaldehyde (PFA) 0.1 M phosphate buffer (PB), pH 7.4 overnight at 4°C, cryoprotected in 30% sucrose, 0.1 M PB and stored at 4°C. The other half was rapidly frozen on dry ice and stored at -80°C. The PFA-fixed brain halves were sectioned into a series of four using a cryostat (CM1950, Leica Biosystems, Deer Park, IL) at 50 μm, which were stored in cryoprotectant (30% sucrose, 1% polyvinyl-pyrrolidone, 0.1 M PB, and ethylene glycol) at 4°C.

### 2.4 Enzyme-linked Immunoassay for plasma corticosterone

Plasma corticosterone levels were measured with an enzyme immunoassay (EIA) previously described (54) with minor modifications. Before running the samples, analytical tests (parallelism and precision tests) were performed with mouse plasma to discard possible interferences and matrix effects. The curve generated by the pooled sample had a displacement parallel to the standard curve. Similarly, precision tests using a pooled sample spiked with corticosterone standards yielded a mean recovery of 120%, indicating no matrix effects. Following the analytical tests, all samples were analyzed using the following methods. Briefly, we pre-coated microplates with 10 µg/ml goat anti-rabbit IgG (111-001-003, Jackson Immunoresearch Laboratories, West Grove, PA) as previously described (55). Corticosterone (16063, Cayman Chemicals, Ann Arbor, MI) was used to prepare the standards. We serially diluted nine standards with EIA buffer, starting at 20 ng/ml. First, we added 50 µl of standards, samples (diluted at 1:20), and control in duplicates to all wells. Immediately after, we added 25 μl of horseradish peroxidase conjugate (diluted at 1:35,000 in EIA buffer) to all wells and 25 μl of the anti-rabbit polyclonal antiserum against corticosterone-3-CMO-BSA (CJM006, diluted at 1:70,000 in EIA buffer) to all wells except non-specific binding wells. The plates were incubated for 1 hour at room temperature, washed 4 x with wash buffer, and developed with 60% 3,3’,5,5’-Tetramethylbenzidine (TMBHK-100, Moss Inc, Pasadena, MD) for 10 minutes. The reaction stopped with 1 N HCl and read using BioTek 800 TS Absorbance Reader. The intra-assay coefficient of variation (CV) was 3.74%, and the inter-assay CV was 6.6%.

### 2.5 Immunofluorescence

Brain sections (one of four series) from vehicle mice and MC-LR mice were simultaneously processed for ΔFOSB immunofluorescence (56–58). The staining conditions were standardized to minimize variability as much as possible. The sections were rinsed 3 x 5 minutes tris-buffered saline and 0.3% Triton-X (TBS-T; 9002-93-1, Fisher Scientific, Pittsburg, PA) on a 2D rotator, and incubated in ΔFOSB rabbit mAB (1:3000, D3S8R, Cell Signaling Technologies, Danvers, MA) diluted on TBS-T and 2% normal goat serum for 2 days at 4°C. Sections were rinsed 3 x 5 minutes with TBS and incubated with biotinylated-goat anti-rabbit (1:600, BA-1000, Vector Laboratories, Burlingame, CA) in TBS for 2 hours at room temperature. Following, sections were rinsed 3 x 5 minutes with TBS and incubated with Alexa Fluor 488-conjugated streptavidin (1:1600, 016-540-084, Jackson Immunoresearch Laboratories, West Grove, PA) in TBS for 1.5 hours at room temperature in dark. Sections were mounted on gelatin-coated glass slides and coverslipped with DABCO mounting medium.

### 2.6 Image analysis

Immunofluorescent photomicrographs of the rostral-caudal PVN (plate 36-39), hippocampal regions: dentate gyrus (DG), CA1, 2 and 3 (plate 42-48) and anterior cortex (plate 25-28) (59) of MC-LR mice and vehicle mice were captured using a 20X objective mounted on an Olympus microscope (BX61, Olympus, Center Valley, PA) fitted with a SC30 color camera (Olympus, Center Valley, PA) connected to a PC. The images (122500 μm^2^) were analyzed with CellSens imaging software (Olympus, Center Valley, PA). We standardized the threshold mask for both vehicle and MC-LR mice to quantify the density of ΔFOSB-immunofluorescent (IF) cells (cells per µm^2^) using CellSens software (Olympus, Center Valley, PA). All the animals were randomized and blinded to minimize the introduction of biases (49, 60).

### 2.7 Quantitative PCR

Sections (150-250 µm) through the hypothalamus, dorsal hippocampus, and anterior motor cortex (61) were used to collect tissue samples using a tissue cannula punch 1 mm diameter (22G29, Integra LifeScience, Mansfield, MA). Total RNA was isolated using TRI-sure (BIO-38033, Meridian Bioscience, Cincinnati, OH) following the manufacturer’s protocol. The concentration of total RNA was determined by using a spectrophotometer to measure the value of absorbance at 260 nm (A260), and the purity of RNA was determined by the ratio of A260 to A280 (Synergy H1 Hybrid Reader, BioTek Instruments, Winooski, VT). Total RNA (500 ng) was converted into the cDNA using LUNAScript RT SuperMix Kit (E3010, New England BioLabs, Ipswich, MA) using oligo-dt primers. qPCR was performed using the gene-specific intron-spanning primers (**Table 1**) and LUNA 480 SYBR Green I Universal qPCR Master Mix (M3003, New England BioLabs, Ipswich, MA). The thermal cycle was set as follows: initial denaturation at 95°C for 2 minutes, followed by 40 cycles of denaturation at 95°C for 15 seconds, annealing at 60°C for 30 seconds, and elongation at 72°C for 30 seconds. Each sample was run in triplicates, and PCR reactions with water (H_2_O) were used as negative controls. Threshold cycle (C_t_) values for the genes of interest were normalized against the housekeeping gene hypoxanthine phosphoribosyltransferase 1 (*Hprt*) value in the same samples (62, 63). The relative fold change in mRNA expression was calculated using 2^-ΔΔCt^ method (64).

**Table 1:** List of all the qPCR primers.

Name	Sequence
*Crh*	FWD-5’-CCTACCAAGGGAGGAGAAGAGAG-3’ REV- 5’-AAGAAATTCAAGGGCTGCGG-3’
*Avp*	FWD-5’-ATCTGCTGCAGCGACGAGAG-3’ REV- 5’-TGTACCAGCCTTAGCAGCAG-3’
*Gr*	FWD-5’-GCAAGTGGAAACCTGCTATGC-3’ REV-5’-AACCGCTGCCAATTCTGACT-3’
*Tnf-α*	FWD-5’-CCCACGTCGTAGCAAACCAC-3’ REV- 5’-TTGAGATCCATGCCGTTGGC-3’
*IL-1β*	FWD-5’-GCCACCTTTTGACAGTGATGAG-3’ REV- 5’-GACAGCCCAFFTCAAAGGTT-3’
*Crp*	FWD-5’-CGGACTTTTGGTCATGAAGACAT-3’ REV- 5’-GTGTGTTGGAGCCTCAGGAA-3’
*Hprt*	FWD-5’-CTCATGGACTGATTATGGACAGGAC-3’ FWD-5’-GCAGGTCAGCAAAGAACTTATAGCC-3’

### 2.8 Statistical analysis

Data was analyzed using Welch’s *t*-tests with treatment as a variable (Sigma plot 14.5, Systat Software, Palo Alto, CA). Differences were considered significant if *p* < 0.05 and indicated as an asterisk.

## 3 Results

### 3.1 Mice body weight and plasma corticosterone levels

**Figure 1:**
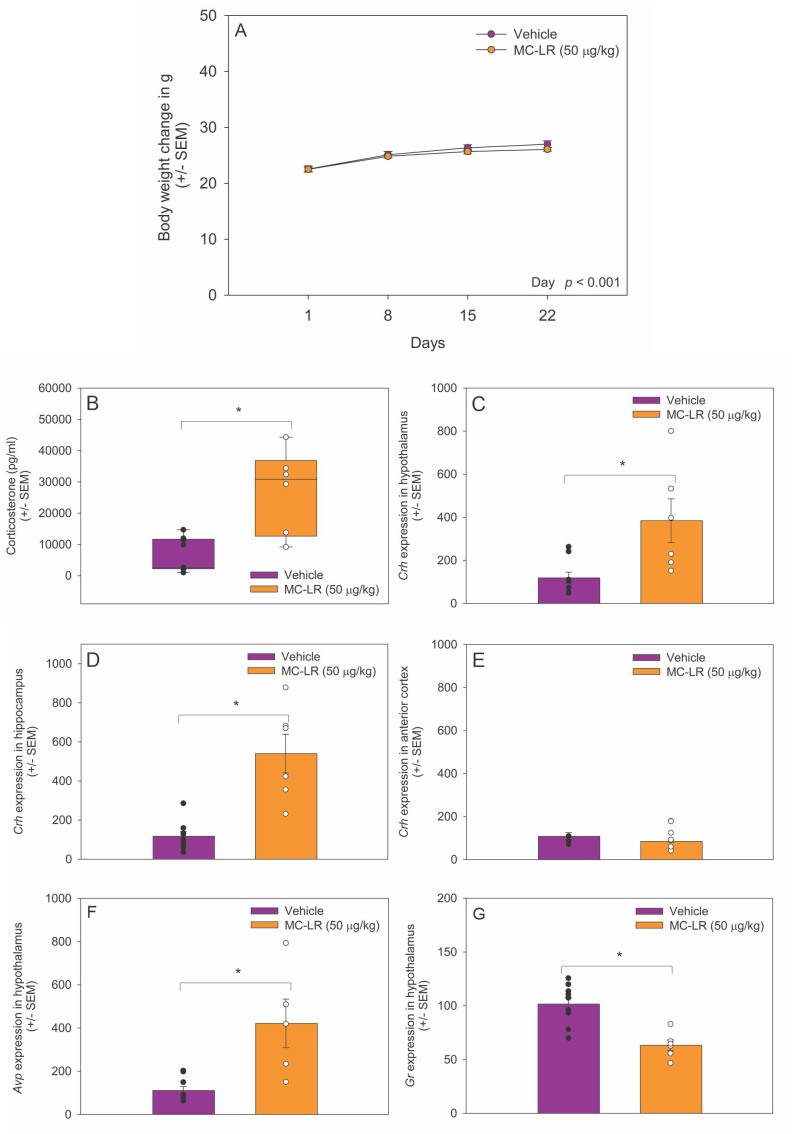
**(A)** Body weight of vehicle and MC-LR mice. Mean body weight did not differ between MC-LR mice and vehicle mice. **(B)** Box plot indicating plasma corticosterone level was higher in MC-LR mice compared to vehicle mice. **(C-E)** Bar graphs indicating that the expression of *Crh* mRNA higher in the hypothalamus and hippocampus of MC-LR mice compared to vehicle mice. **(F)** Bar graph indicating that the expression of *Avp* mRNA in the hypothalamus was higher in MC-LR mice compared to vehicle mice. **(G)** Bar graph indicating that the expression of *Gr* mRNA in the hypothalamus was lower MC-LR mice compared to vehicle mice. Values are represented as mean ± standard error, n = 9 for vehicle mice and n = 6 for MC-LR mice. * Indicates *p* < 0.05.

We measured the mouse body weight every day during the experimental period. No significant changes in body weight were detected between MC-LR mice and vehicle mice (**Figure 1A**). In contrast, plasma corticosterone levels were significantly higher in MC-LR mice compared to vehicle mice (*t*_(13)_ = -3.637, *p* < 0.05) (**Figure 1B**).

### 3.2 MC-LR affected hypothalamic Crh, Avp, and Gr mRNA expression

The expression of *Crh* mRNA was higher in the hypothalamus of MC-LR mice compared to vehicle mice (*t*_(13)_ = -4.877, *p* < 0.05) (**Figure 1C**). The expression of *Crh* mRNA was also higher in the hippocampus of MC-LR mice compared to vehicle mice (*t*_(13)_ = -4.979, *p* < 0.05) (**Figure 1D**). No significant difference in *Crh* mRNA expression was found in the anterior motor cortex between MC-LR mice and vehicle mice (**Figure 1E**). Moreover, *Avp* mRNA expression was higher in hypothalamus of MC-LR mice compared to vehicle mice (*t*_(13)_ = -3.637, *p* < 0.05) (**Figure 1F**). In contrast, the expression of *Gr* mRNA in the hypothalamus was significantly lower in MC-LR mice compared to vehicle mice (*t*_(13)_ = 4.389, *p* < 0.05) (**Figure 1G**).

### 3.3 MC-LR effects on ΔFOSB-IF cell density in the PVN, hippocampus, and anterior motor cortex

**Figure 2:**
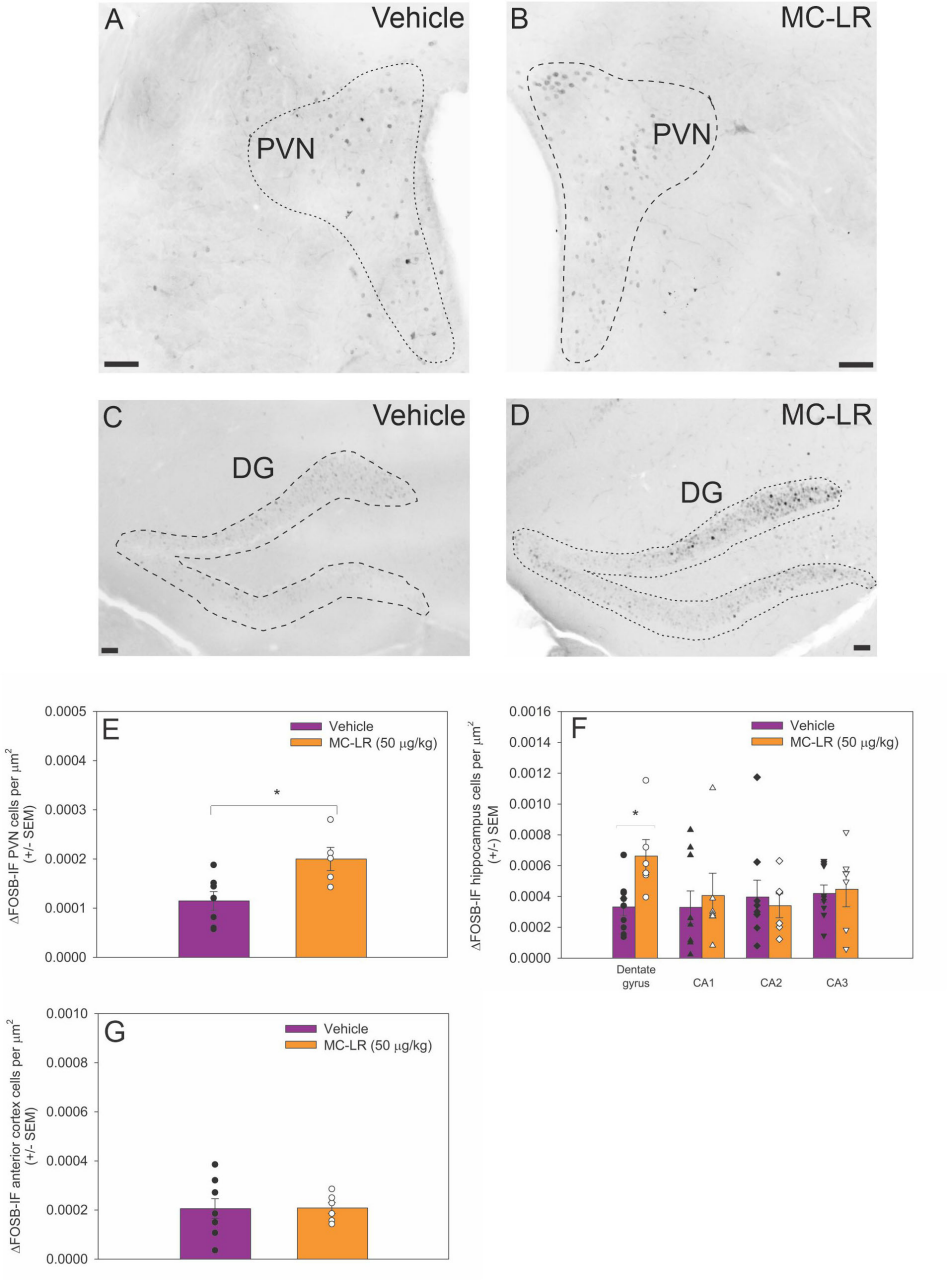
**(A, B)** Photomicrographs of ΔFOSB immunofluorescent cells in the PVN and **(C, D)** DG of vehicle and MC-LR mice. For clarity, photomicrographs were converted to grey scale and inverted using Corel PhotoPaint. **(E)** Bar graph indicating that the density of ΔFOSB-IF PVN cells was higher in MC-LR mice compared to vehicle mice. **(F)** Bar graph indicating that the density of ΔFOSB-IF DG cells was higher in MC-LR mice compared to vehicle mice. The density of ΔFOSB-IF cells in the CA 1–3 did not differ between MC-LR mice and vehicle mice. **(G)** Bar graph indicating that the density of ΔFOSB-IF anterior cortex cells did not differ between MC-LR mice and vehicle mice. Scale bar represents 100 µm. Values are represented as mean ± standard error, n = 9 for vehicle mice and n = 6 for MC-LR mice. * Indicates *p* < 0.05.

The ΔFOSB-IF cell density in the PVN was higher in MC-LR mice compared to vehicle mice (*t*_(10)_ = -2.851, *p* < 0.05) (**Figures 2A, B, E**). Similarly, ΔFOSB-IF cell density in the DG was higher in MC-LR mice compared to vehicle mice (*t*_(13)_ = -2.980, *p* < 0.05) (**Figures 2C, D, F**), whereas ΔFOSB-IF cell density did not differ in CA 1, 2 and 3 regions between MC-LR mice and vehicle mice. No significant difference in ΔFOSB-IF cell density was detected in anterior cortex between MC-LR mice and vehicle mice (**Figure 2G**).

### 3.4 MC-LR did not affect inflammation markers in the hypothalamus, liver, or spleen

**Figure 3:**
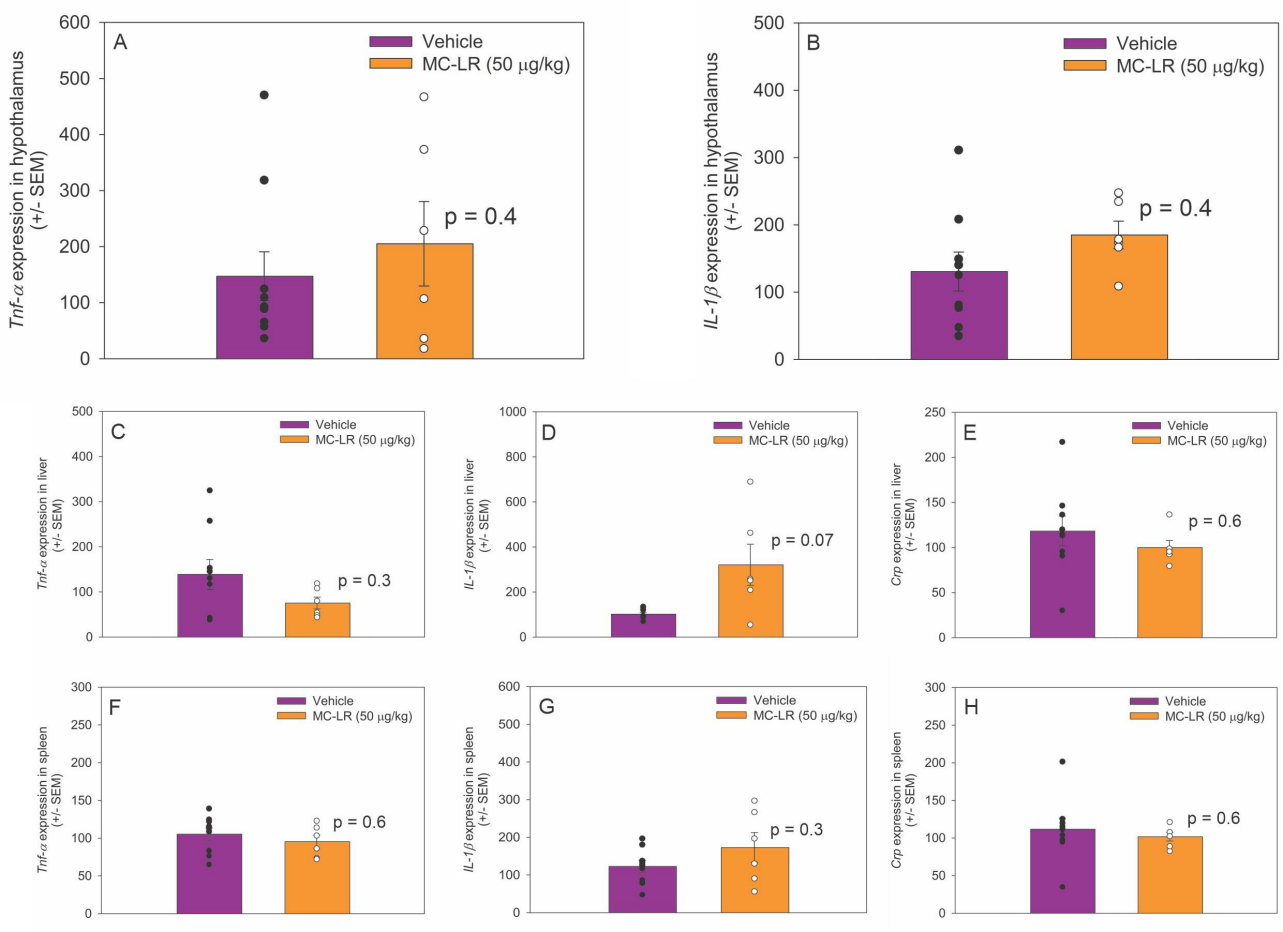
**(A, B)** Bar graphs indicating that hypothalamic *Tnf-α* and *IL-1β* mRNA expression did not differ between MC-LR mice and vehicle mice. **(C-E)** Bar graphs indicating that liver *Tnf-α, IL-1β and Crp* mRNA expression did not differ between MC-LR mice and vehicle mice. **(F-H)** Bar graphs indicating that spleen *Tnf-α, IL-1β and Crp* mRNA expression did not differ between MC-LR mice and vehicle mice. Values are represented as mean ± standard error, n = 9 for vehicle mice and n = 6 for MC-LR mice. * Indicates *p* < 0.05.

In the hypothalamus, there was no significant difference in *Tnf-α* (*t*_(13)_ = -0.9, *p* = 0.4) and *IL-1β* (*t*_(13)_ = -1.0, *p* = 0.3) mRNA expression between MC-LR mice and vehicle mice (**Figures 3A, B**). *Crp* mRNA was not detected in the hypothalamus of MC-LR mice or vehicle mice. In the liver, *Tnf-α* (*t*_(13)_ = 1.125, *p* = 0.30), IL*-1β* (*t*_(13)_ = -2.1, *p* = 0.07), and *Crp* (*t*_(13)_ = 0.6, *p* = 0.6) mRNA expression did not differ between MC-LR mice and vehicle mice (**Figures 3C-E**). In the spleen, *Tnf-α* (*t*_(13)_ = 0.6, *p* = 0.6), *IL-1β* (*t*_(13)_ = -1.1, *p* = 0.3), and *Crp* (*t*_(13)_ = 0.5, *p* = 0.6) mRNA expression did not differ between MC-LR mice and vehicle mice (**Figures 3F-H**).

## 4 Discussion

Here, we report that short-term (i.e., every 48 hours for 21 days) ingestion of sublethal levels of MC-LR increased plasma corticosterone levels, hypothalamic *Crh* and *Avp* mRNA expression, and ΔFOSB immunoreactivity in the PVN and hippocampal dentate gyrus, which are indicative of HPA activation. These results in mice are in line with earlier long-term sublethal MC-LR exposure studies in zebrafish and rats (50, 65). In contrast, hypothalamic *Gr* mRNA expression, which is known to facilitate the negative feedback, was significantly lower in MC-LR mice. MC-LR did not cause a significant inflammatory response in the hypothalamus, liver, or spleen, suggesting that the oral MC-LR dose used in this study did not elicit a significant immune response. Together, these observations led to the conclusion that short-term ingestion of sublethal levels of MC-LR results in HPA hyperactivity, partly due to hypothalamic dysregulation of negative feedback.

In contrast to our short-term sublethal MC-LR ingestion paradigm in male mice, earlier acute MC-LR dosing studies in adult male and female rats showed that a single intraperitoneal dose of MC-LR reduced HPA activity in both males and females one day later (33, 34). These differences may be due to the MC-LR administration route and length of administration. Studies in mice showed that MC-LR is far more toxic when administered intraperitoneally than orally. Toxicological studies in mice found intraperitoneal MC-LR LD50 to be 50 μg/kg, whereas oral MC-LR toxicity was 30–100 times less (16, 66–68). Alternatively, MC-LR dosing duration may also have played a role; for instance, in contrast to the aforementioned acute male and female rat studies (33, 34), a recent study in male rats found that intraperitoneal administration of sublethal MC-LR levels for 6 weeks also increased plasma corticosterone levels (50). Therefore, the route and length of MC-LR exposure must be considered when evaluating and determining MC-LR toxicity on HPA function in mammals.

Short-term sublethal MC-LR ingestion increased plasma corticosterone levels, which confirmed that ingested MC-LR can activate the stress axis. Based on this observation, we hypothesized that short-term sublethal ingestion MC-LR may have activated hypothalamic neuroendocrine cells that control the stress response. In support, we demonstrated that hypothalamic *Crh* and *Avp* mRNA expression were elevated in MC-LR mice. Moreover, ΔFOSB immunoreactivity in PVN and hippocampal dentate gyrus was higher in MC-LR mice than in vehicle mice. The results clearly demonstrate that elevated corticosterone levels following short-term sublethal MC-LR ingestion are a direct consequence of the activation of hypothalamic and hippocampal neurons responsible for regulating HPA activity. The elevated hypothalamic *Avp* mRNA expression may indicate that short-term sublethal MC-LR ingestion in our paradigm was sufficient to stimulate hypothalamic *Avp* mRNA, which in previous rat and human studies was shown to be indicative of prolonged stress and potentiation of CRH-dependent activation of the stress axis (37, 38, 43, 44, 69, 70).

The stimulatory central limb of the stress axis (i.e., CRH and AVP) is kept in balance by circulating corticosterone, which provides negative feedback to the pituitary, hypothalamus, and hippocampus to return the animal to homeostatic physiological conditions (35–42). Generally, glucocorticoid negative feedback is mediated by two corticosteroid receptor types: mineralocorticoid receptor (MR) and GR, which reside in hypothalamic and hippocampal neurons and pituitary cells. Because of their high affinity for glucocorticoids, MRs are thought to regulate basal hormone secretion. In contrast, GRs, which exhibit an approximately 10-fold lower affinity for glucocorticoids, are thought to turn off the HPA axis and return stress-responsive glucocorticoid elevations to baseline (37, 71, 72). Based on our observation demonstrating that hypothalamic *Gr* mRNA expression was decreased in MC-LR mice, we infer that MC-LR may have attenuated glucocorticoid-dependent negative feedback. Alternatively, the cellular actions of MC-LR, a potent inhibitor of protein phosphatase PP1 and PP2A (29, 30, 73), have been shown to cause GR hyperphosphorylation, which may signal increased GR degradation (74). Also, the hyperphosphorylation of GR might lead to reduced glucocorticoid sensitivity (75).

It is unclear whether ingested MC-LR activates hypothalamic and hippocampal neurons directly or indirectly to trigger the stress response. However, previous studies showed that following passive absorption by the small intestines, MC-LR passes into the portal vein to travel by the blood to other organs, such as the brain. Microcystin-LR can be quickly taken up by brain cells due to the wide-spread presence of transmembrane OATPs in the blood-brain-barrier endothelial cells and blood-cerebrospinal fluid barrier epithelial cells (76), and enter brain cells (22, 77), including neuroendocrine cells (78, 79) as demonstrated using *in vitro* neuron cell models. These studies indicate that MC-LR may be able to enter the brain. However, more in depth cell-specific studies are needed to assess whether this is the case.

In the current study, we found that oral ingestion of MC-LR activated the HPA axis, in contrast to the central and peripheral inflammatory response. These results indicate that while short-term ingestion of sublethal levels of MC-LR did not cause significant activation of the inflammatory system, it was sufficient to activate the HPA axis and therefore may potentially contribute to stress-related mental health conditions, such as anxiety and depression. However, further investigations are needed to elucidate the molecular and cellular mechanisms of the impact of MC-LR on the HPA axis.

## Data Availability

The original contributions presented in the study are included in the article/Supplementary Material. Further inquiries can be directed to the corresponding author.
