# Supplementary figures and images for: Short-term ingestion of sublethal microcystin levels disrupts stress response in male mice
Source: Front Endocrinol (Lausanne). 2025 May 26;16:1568923. doi: 10.3389/fendo.2025.1568923 (PMC12146875; doi:10.3389/fendo.2025.1568923)

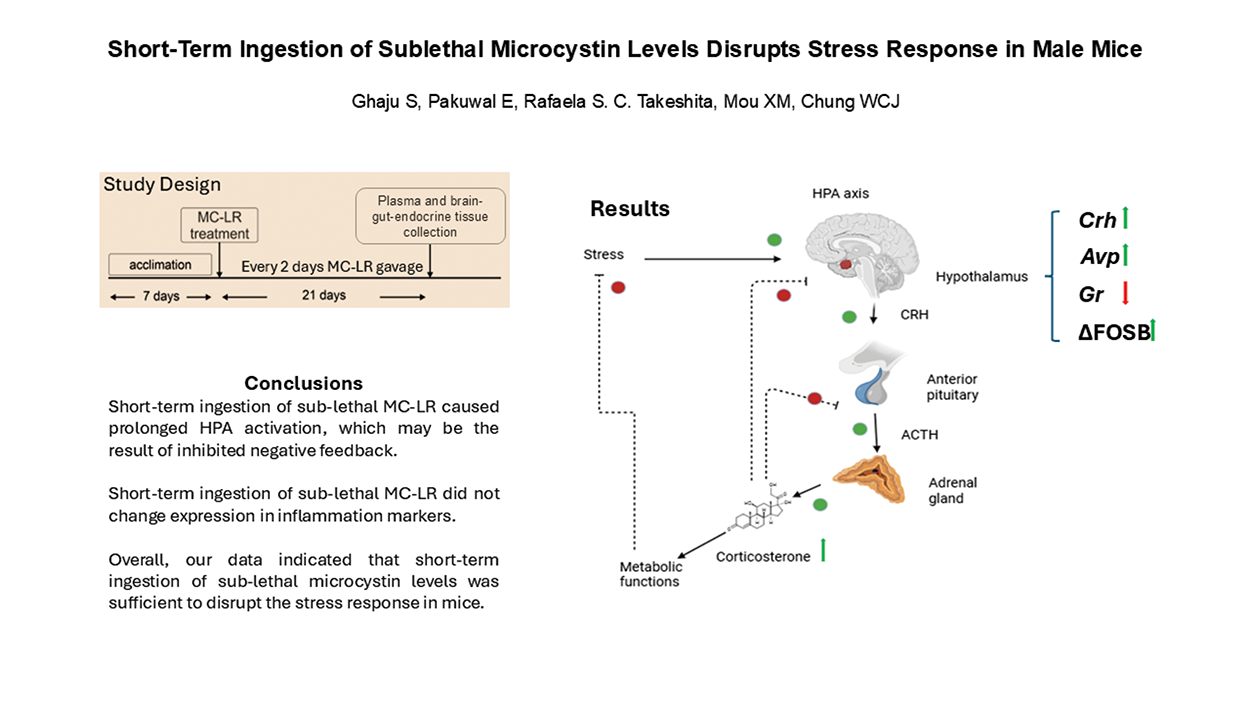

Supplement: Supplementary file 1 [file Image1.tif]
